# Timing of oral anticoagulants initiation for atrial fibrillation after acute ischemic stroke: A systematic review and meta-analysis

**DOI:** 10.1177/23969873241251931

**Published:** 2024-05-14

**Authors:** Lina Palaiodimou, Maria-Ioanna Stefanou, Aristeidis H Katsanos, Gian Marco De Marchis, Diana Aguiar De Sousa, Jesse Dawson, Mira Katan, Theodore Karapanayiotides, Konstantinos Toutouzas, Maurizio Paciaroni, David J Seiffge, Georgios Tsivgoulis

**Affiliations:** 1 Second Department of Neurology, “Attikon” University Hospital, School of Medicine, National and Kapodistrian University of Athens, Athens, Greece; 2 Department of Medicine (Neurology), McMaster University/Population Health Research Institute, Hamilton, Canada; 3 Department of Clinical Research, University of Basel, Basel, Switzerland; 4 Department of Neurology and Stroke Center, Kantonsspital St. Gallen, Switzerland; 5 Department of Neurosciences (Neurology), Hospital de Santa Maria, University of Lisbon, Lisbon, Portugal; 6 Institute of Cardiovascular and Medical Sciences, University of Glasgow, Glasgow, UK; 7 Department of Neurology, University Hospital of Zurich, Neuroscience Center Zurich, University of Zurich, Zurich, Switzerland; 8 Department of Neurology, University Hospital and University of Basel, Basel, Switzerland; 9 Second Department of Neurology, School of Medicine, Faculty of Health Sciences, AHEPA University General Hospital, Aristotle University of Thessaloniki, Thessaloniki, Greece; 10 First Department of Cardiology, National and Kapodistrian University of Athens, “Hippokration” Hospital, Athens, Greece; 11 Stroke Unit and Division of Cardiovascular Medicine, University of Perugia, Italy; 12 Department of Neurology, Inselspital, Bern University Hospital, University of Bern, Bern, Switzerland

**Keywords:** Acute ischemic stroke, atrial fibrillation, oral anticoagulants, meta-analysis, secondary prevention, intracerebral hemorrhage

## Abstract

**Introduction:**

There is a longstanding clinical uncertainty regarding the optimal timing of initiating oral anticoagulants (OAC) for non-valvular atrial fibrillation following acute ischemic stroke. Current international recommendations are based on expert opinions, while significant diversity among clinicians is noted in everyday practice.

**Methods:**

We conducted an updated systematic review and meta-analysis including all available randomized-controlled clinical trials (RCTs) and observational cohort studies that investigated early versus later OAC-initiation for atrial fibrillation after acute ischemic stroke. The primary outcome was defined as the composite of ischemic and hemorrhagic events and mortality at follow-up. Secondary outcomes included the components of the composite outcome (ischemic stroke recurrence, intracranial hemorrhage, major bleeding, and all-cause mortality). Pooled estimates were calculated with random-effects model.

**Results:**

Nine studies (two RCTs and seven observational) were included comprising a total of 4946 patients with early OAC-initiation versus 4573 patients with later OAC-initiation following acute ischemic stroke. Early OAC-initiation was associated with reduced risk of the composite outcome (RR = 0.74; 95% CI:0.56–0.98; *I*^2^ = 46%) and ischemic stroke recurrence (RR = 0.64; 95% CI:0.43–0.95; *I*^2^ = 60%) compared to late OAC-initiation. Regarding safety outcomes, similar rates of intracranial hemorrhage (RR = 0.98; 95% CI:0.57–1.69; *I*^2^ = 21%), major bleeding (RR = 0.78; 95% CI:0.40–1.51; *I*^2^ = 0%), and mortality (RR = 0.94; 95% CI:0.61–1.45; *I*^2^ = 0%) were observed. There were no subgroup differences, when RCTs and observational studies were separately evaluated.

**Conclusions:**

Early OAC-initiation in acute ischemic stroke patients with non-valvular atrial fibrillation appears to have better efficacy and a similar safety profile compared to later OAC-initiation.

## Graphical abstract

**Graphical abstract:**
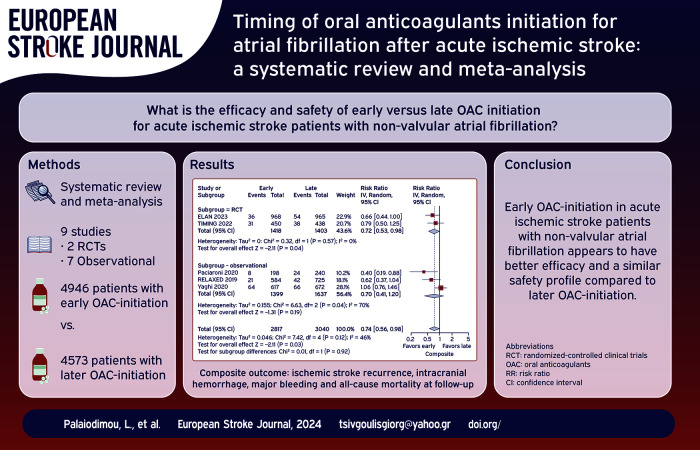

## Introduction

Oral anticoagulants (OAC), including vitamin K antagonists and non-vitamin K antagonist oral anticoagulants (NOACs), are indicated for the secondary stroke prevention of patients with non-valvular atrial fibrillation.^1^ However, there is a longstanding clinical uncertainty regarding the optimal timing of initiating OACs after an acute ischemic stroke among patients with non-valvular atrial fibrillation.^2^ This uncertainty reflects the challenge to preserve the delicate equilibrium between thrombosis and hemostasis: clinicians are wavering that early OAC initiation may prevent early stroke recurrence,^3^ yet at a potential cost of increased (intracranial) bleeding early after acute ischemic stroke.^2^ Available international guidelines, including the American Heart Association/American Stroke Association,^4^ the European Society of Cardiology,^1,5^ and the European Stroke Organization guidelines,^6^ underscore the paucity of robust data to guide OAC (re)initiation and its optimal timing.

In a previous systematic review and meta-analysis performed by our group,^7^ similar rates of ischemic stroke recurrence, intracranial hemorrhage and all-cause mortality were shown for patients that initiated OACs within the first week versus within 2 weeks after acute ischemic stroke (test for subgroup differences *p* = 0.1677; *p* = 0.8941; and *p* = 0.7786 accordingly). Apart from including mostly observational data, this study was also limited by the fact that the results were based on subgroup comparisons in single-arm meta-analyses. Despite those shortcomings, there was a clear signal of similar efficacy and safety between early and late OAC initiation that warranted further research. Indeed, since the publication of this study, two randomized-controlled clinical trials (RCTs) comparing head-to-head early versus late OAC initiation presented their results, the Early versus Late initiation of direct oral Anticoagulants in post-ischemic stroke patients with atrial fibrillatioN (ELAN) and the Timing of Oral Anticoagulant Therapy in Acute Ischemic Stroke with Atrial Fibrillation (TIMING) trials,^8,9^ mandating an updated systematic review and meta-analysis.

In view of the former considerations, we sought to conduct an updated systematic review and meta-analysis with the aim to assess the efficacy and safety of early versus late OAC initiation for acute ischemic stroke patients with non-valvular atrial fibrillation.

## Methods

### Standard protocol approvals, registrations, and patient consents

The pre-specified protocol of the present systematic review and meta-analysis has been registered in the International Prospective Register of Ongoing Systematic Reviews PROSPERO (registration ID: CRD42024507385) and is reported according to the updated Preferred Reporting Items for Systematic Reviews and Meta-Analyses (PRISMA) guidelines.^10^ No ethical board approval or written informed consent by the patients were required due to the study design (systematic review and meta-analysis).

### Data sources, searches, and study selection

Following the PICO format, a systematic literature search was conducted to identify available RCTs and observational cohort studies (including individual patient-data pooled analysis of cohorts) that evaluated adult patients with acute ischemic stroke and non-valvular atrial fibrillation (*P*: population) initiating OACs at an early stage post acute ischemic stroke (early as defined in each study; *I*: intervention) compared to initiating OACs at a later stage (*C*: comparator). Reporting of ischemic and hemorrhagic events and mortality at follow-up (*O*: outcome) was required for studies to be considered eligible for inclusion. The literature search was performed independently by two reviewers (LP, and MIS). We searched MEDLINE, and Scopus, using search strings that included the following terms: “stroke,” “atrial fibrillation,” “oral anticoagulants,” and “initiation.” No language or other restrictions were applied. Our search spanned from inception of each database to January 15^th^, 2024. We additionally searched reference lists of published articles and international conference abstracts manually, to ensure the comprehensiveness of bibliography.

Non-controlled studies, case series and case reports were excluded. Commentaries, editorials, and narrative reviews were also discarded. All retrieved studies were independently assessed by the two reviewers (LP, and MIS) and any disagreements were resolved after discussion with a third tie-breaking evaluator (GT).

### Quality control, bias assessment, and data extraction

Eligible studies were subjected to quality control and bias assessment employing the Cochrane Collaboration tool (RoB 2)^11^ for RCTs and the Risk Of Bias In Non-randomized Studies of Interventions (ROBINS-I)^12^ tool for cohort studies. Quality control and bias assessment was conducted independently by three reviewers (LP, MIS, and AHK), and disagreements were settled by consensus after discussion with the corresponding author (GT).

Data extraction was performed on structured forms, including trial names, patient sample, patients’ characteristics, time windows of OAC initiation, OAC types used and outcomes of interest.

### Outcomes

The primary outcome of interest was the composite of ischemic and hemorrhagic events and mortality at follow-up. Secondary outcomes included the components of the composite outcome (ischemic stroke recurrence, intracranial hemorrhage, major bleeding, and all-cause mortality).

### Statistical analysis

For the pairwise meta-analysis, we calculated for each dichotomous outcome of interest the corresponding risk ratios (RR) with 95% confidence interval (95% CI) for the comparison of outcome events among patients initiating OACs early versus later post acute ischemic stroke. Subgroup analysis was performed stratified by study design, that is, RCTs versus observational studies, and by the different time window defining early OAC initiation (in days after acute ischemic stroke). Comparison of the baseline characteristics to assess the balance between the two arms was performed using odds ratios (OR) for dichotomous variables and the mean difference for continuous variables. For studies reporting continuous outcomes in median values and corresponding interquartile ranges we estimated the sample mean and standard deviation using the quantile estimation method.^13^ Since in our previous meta-analysis, a signal of increased efficacy favoring NOACs compared to vitamin K antagonists was shown both in the early and in the late treatment window, a sensitivity analysis was conducted by including the studies in which the patients were prescribed NOACs only. The random-effects model (DerSimonian and Laird) was used to calculate the pooled estimates.^14^ Heterogeneity was assessed with the *I*^2^ and Cochran *Q* statistics. For the qualitative interpretation of heterogeneity, *I*^2^ values <25%, between 25% and 50% and >50% were considered to represent low, moderate, and significant heterogeneity, respectively. The significance level for the *Q* statistic was set at 0.1. Publication bias across individual studies was assessed when more than four studies were included in the analysis of the outcomes of interest, using both funnel plot inspection and the Egger et al.’s linear regression test,^15^ and the equivalent *z* test for each pooled estimate with a two-tailed *p* value < 0.05 was considered statistically significant. All meta-analyses were performed as described in the prespecified protocol (registration ID: CRD42024507385), with no deviations noted. The above statistical analyses were performed using the R software version 3.5.0 (package: meta).^16^ Finally, a trial sequential analysis was performed for the primary outcome of interest by including RCTs only, to better inform future enrollment in ongoing studies, using the TSA software (0.9.5.10 Beta, The Copenhagen Trial Unit, Copenhagen, Denmark).^17^

### Data availability statement

All data generated or analyzed during this study are included in this article and its supplementary information files.

## Results

### Literature search and included studies

**Figure 1.**
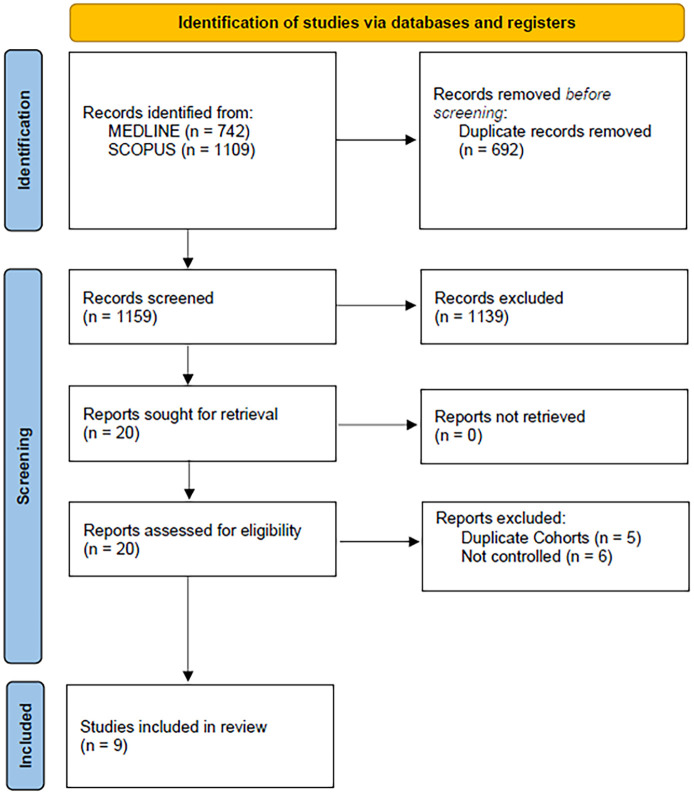
Flow chart of the systematic review.

The flow diagram for the selection and inclusion of studies in this systematic review is presented in Figure 1. After excluding duplicates, the systematic literature database search yielded a total of 1159 records. Following the initial screening process, the full texts of 20 records were retrieved. After reading the full-text articles, 11 records were further excluded. Finally, we included nine eligible studies (two RCTs and seven observational studies)^8,9,18–24^ in the systematic review and meta-analysis, comprising a total of 4946 patients with early OAC-initiation versus 4573 patients with later OAC-initiation. The characteristics of the included studies are summarized in Table 1.

**Table 1.** Characteristics of studies included in the systematic review and meta-analysis.

Study	Design	Country	Time window of initiation		Follow-up	Type of OAC	
			Early	Late		VKA	NOAC
Al Bakr et al.^18^	Observational	Saudi Arabia	⩽6 days	>6 days	3 months	+	+
De Marchis et al.^19^	Observational (IPDPA)	Seven European and Japanese prospective observational cohort studies	⩽5 days	>5 days	At least 3 months	−	+
ELAN^8^	RCT	International	within 48 hours after a minor or moderate stroke or on day 6 or 7 after a major stroke	day 3 or 4 after a minor stroke, day 6 or 7 after a moderate stroke, or day 12, 13, or 14 after a major stroke	3 months	−	+
Kimura et al.^20^	Observational	Japan	⩽7 days	>7 days	3 months	+	+
Matos-Ribeiro et al.^21^	Observational	Portugal	⩽4 days	>4 days	3 months	+	+
Paciaroni et al.^22^	Observational	International	⩽7 days	>7 days	3 months	+	+
RELAXED^23^	Observational	Japan	⩽2 days	>2 days	3 months	−	+
TIMING^9^	RCT	Sweden	⩽4 days	>4 days	3 months	−	+
Yaghi et al.^24^	Observational	United States	⩽3 days	>3 days	3 months	+	+

OAC: oral anticoagulant; VKA: vitamin K antagonists; NOAC: non-vitamin K antagonist oral anticoagulant; IPDA: individual patient data pooled analysis.

### Quality control of included studies

The risk of bias among the included RCTs was assessed by the Cochrane risk-of-bias (RoB 2) tool^11^ and is presented in eFigures 1 to 2. Both studies were of excellent quality regarding the randomization and reporting processes. However, both studies were limited since patients and investigators were not blinded to the interventions. Furthermore, there was a minor bias due to missing data in the ELAN trial,^8^ while outcome assessment was not reported to be blinded in the TIMING trial.^9^

The risk of bias among the included observational studies was assessed by the Risk Of Bias In Non-randomized Studies of Interventions (ROBINS-I) tool^12^ and is presented in eFigures 3 to 4. All studies presented bias in classification of interventions, since patients with more severe strokes tended to initiate OACs in the later treatment window. Measurement of outcomes was not stated to be blinded in any of the studies, resulting in moderate bias. Finally, moderate bias due to selection of participants was noted in the study of Paciaroni et al.,^22^ since in this specific study only patients with posterior circulation acute ischemic stroke were included.

### Quantitative analyses

Regarding the baseline characteristics (eFigures 5–11), there were no significant differences in age (mean difference: 0.10; 95% CI: −1.80–2.01 years), sex (OR: 0.99; 95% CIL 0.90–1.08), CHA_2_DS_2_VASc (mean difference: 0.22; 95% CI: −0.25 – 0.68) and HASBLED scores (mean difference: 0; 95% CI: −0.08 – 0.08), proportion of patients receiving acute reperfusion treatments (OR: 0.69; 95% CI: 0.45–1.04), and proportion of patients with small infarct acute ischemic stroke (OR: 2.72; 95% CI: 0.70–10.57). Yet, patients initiating early OACs had significantly lower National Institutes of Health Stroke Scale (NIHSS) scores at admission compared to patients with a later OAC initiation (mean difference: −3.10; 95% CI: −5.36–−0.84).

**Figure 2.**
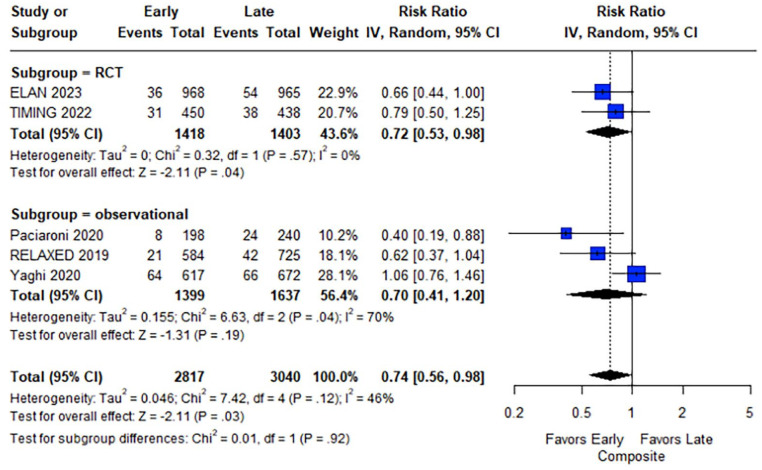
Forest plot presenting the risk ratio of the composite outcome at follow-up among the patients in the early versus late group of oral anticoagulant initiation.

**Figure 3.**
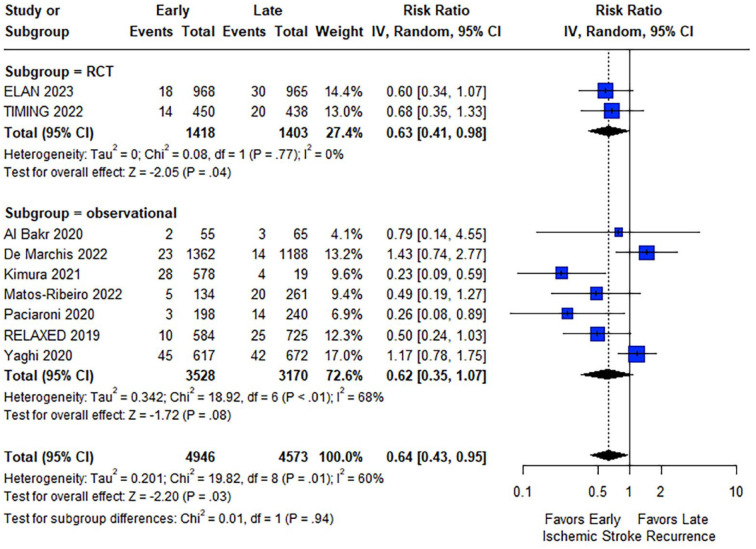
Forest plot presenting the risk ratio of ischemic stroke recurrence at follow-up among the patients in the early versus late group of oral anticoagulant initiation.

**Figure 4.**
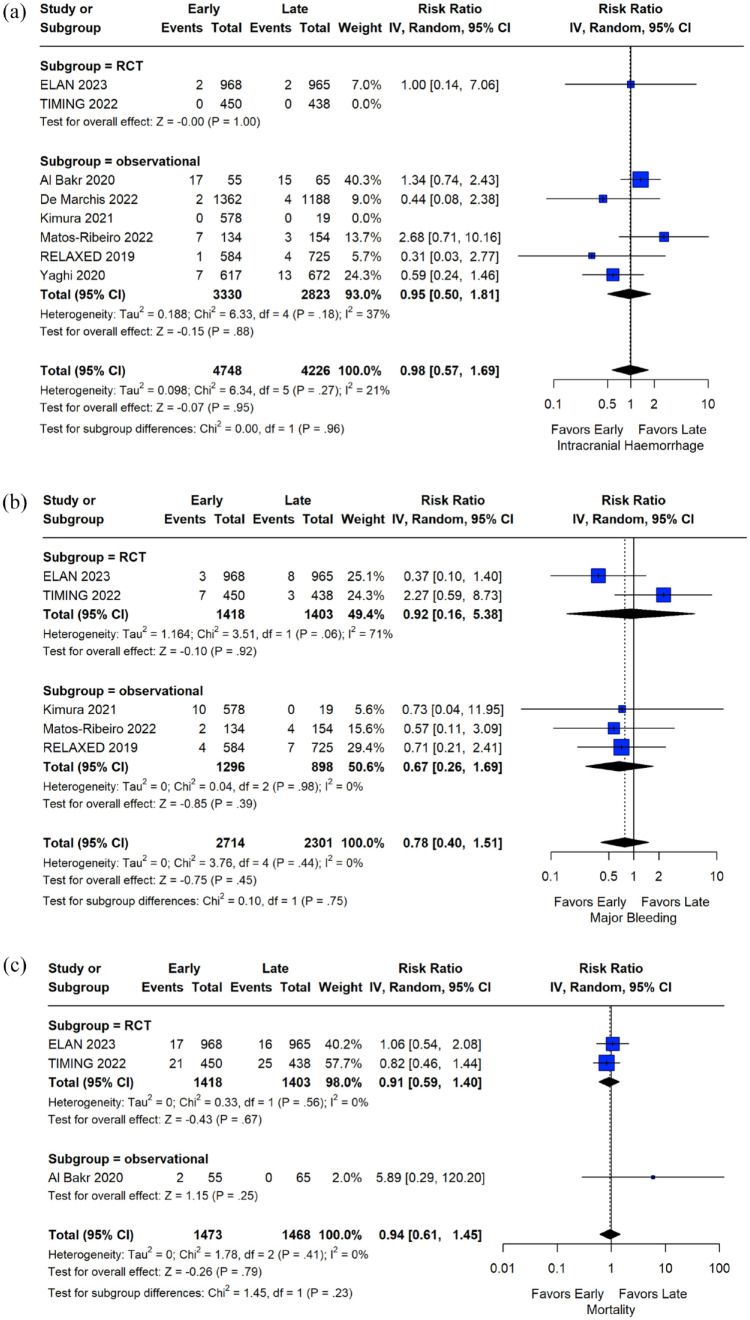
Analysis of safety outcomes. Forest plot presenting the risk ratio of intracranial hemorrhage (Panel A), major bleeding (Panel B), and all-cause mortality (Panel C) at follow-up among the patients in the early versus late group of oral anticoagulant initiation.

Regarding the outcomes of interest, patients initiating OACs at an early stage after acute ischemic stroke had lower rates of the composite outcome at follow-up compared to patients with a later OAC initiation (RR: 0.74; 95% CI: 0.56–0.98; *I*^2^ = 46%; *p* for Cochran *Q* = 0.12; Figure 2). Reduced ischemic stroke recurrence was also shown for patients with early OAC initiation (RR: 0.64; 95% CI: 0.43–0.95; *I*^2^ = 60%; *p* for Cochran *Q* = 0.01; Figure 3). Regarding safety outcomes, similar rates of intracranial hemorrhage (RR: 0.98; 95% CI: 0.57–1.69; *I*^2^ = 21%; *p* for Cochran *Q* = 0.27), major bleeding (RR: 0.78; 95% CI: 0.40–1.51; *I*^2^ = 0%; *p* for Cochran *Q* = 0.44) and all-cause mortality (RR: 0.94; 95% CI: 0.61–1.45; *I*^2^ = 0%; *p* for Cochran *Q* = 0.41) were observed between the two groups (Figure 4). An overview of analyses for all primary and secondary, is summarized in Table 2. There were no subgroup differences, when RCTs and observational studies were separately evaluated. Importantly, early OAC initiation was also associated with reduced risk of the composite outcome (RR: 0.72; 95% CI: 0.53–0.98; *I*^2^ = 0%; *p* for Cochran *Q* = 0.57) and recurrent ischemic stroke (RR: 0.63; 95% CI: 0.41–0.98; *I*^2^ = 0%; *p* for Cochran *Q* = 0.77) without any increase in intracranial hemorrhage (RR = 1.00; 95% CI: 0.14–7.06), major bleeding (RR = 0.92; 95% CI: 0.16–5.38; *I*^2^ = 71%; *p* for Cochran *Q* = 0.06) or mortality (RR = 0.91; 95% CI: 0.59–1.40; *I*^2^ = 0%; *p* for Cochran *Q* = 0.56) in the subgroup of RCTs. When the studies were stratified according to the different time windows for early OAC initiation, no subgroup differences emerged for any of the outcomes (all *p*-values for subgroup differences ⩾0.05; eFigures 12–16). Sensitivity analysis, that was restricted to the studies that included patients specifically initiating NOACs, confirmed the previous results (eFigures 17–21), with one exception: although ischemic stroke recurrence was lower among patients with early NOAC initiation, this difference did not reach statistical significance (RR: 0.74; 95% CI: 0.47–1.15).

**Table 2.** Overview of analyses for the outcomes of interest.

Variable	Effect		
	Risk ratio (95% Confidence interval)	*p* value	*I*^2^; *p* for Cochran *Q*
Primary outcome			
Composite outcome (all studies)	0.74 (0.56–0.98)	0.03	46%; 0.12
Composite outcome (RCTs)	0.72 (0.53–0.98)	0.04	0%; 0.57
Secondary outcomes			
Ischemic stroke recurrence (all studies)	0.64 (0.43–0.95)	0.03	60%; 0.01
Ischemic stroke recurrence (RCTs)	0.63 (0.41–0.98)	0.04	0%; 0.77
Intracranial hemorrhage (all studies)	0.98 (0.57–1.69)	0.95	21%; 0.27
Intracranial hemorrhage (RCTs)	1.00 (0.14–7.06)	>0.99	NA
Major bleeding (all studies)	0.78 (0.40–1.51)	0.45	0%; 0.44
Major bleeding (RCTs)	0.92 (0.16–5.38)	0.92	71%; 0.06
All-cause mortality (all studies)	0.94 (0.61–1.45)	0.79	0%; 0.41
All-cause mortality (RCTs)	0.91 (0.59–1.40)	0.67	0%; 0.56

RCTs: randomized-controlled clinical trials; NA: not applicable.

Although no publication biases were detected during evaluation for the safety outcomes, that is, intracranial hemorrhage and major bleeding (evaluation for mortality was not performed since less than four studies were included in the main analysis), significant asymmetry was noted when the composite outcome and ischemic stroke recurrence were evaluated, most probably due to small-study effects (eFigures 22–25).

Finally, we performed a trial sequential analysis for the primary outcome of interest by including RCTs only, calculating a power of 90% to detect a relative risk reduction of 30%. According to the generated graph (eFigure 26), the last point of *Z*-curve is outside the conventional test boundary but within the monitoring boundaries, while the inner wedge is not reached. The required information size has been calculated at 3633 patients, that is, an additional 812 patients from a future RCT.

## Discussion

The present meta-analysis shows that initiating OACs early after acute ischemic stroke in patients with non-valvular atrial fibrillation is associated with lower rates of the composite of ischemic and hemorrhagic events and mortality at follow-up (RR: 0.74; 95% CI: 0.56–0.98) as a net benefit outcome, and of ischemic stroke recurrence in particular (RR: 0.64; 95% CI: 0.43–0.95), while rates of intracranial hemorrhage, major bleeding and all-cause mortality, as separately assessed, are similar between the two groups.

Furthermore, the subgroup analysis stratifying for study design confirmed the above results. The synthesis of high-quality data derived from RCTs only, that presented only minor risk of bias, showed increased efficacy of early versus later OAC initiation, while safety outcomes remained similar between the groups. While the evidence supports a shift toward earlier OAC initiation, the precise definition of “early” remains a subject of debate. For that reason, a separate subgroup analysis was also performed to assess whether the time window that defined early OAC initiation interferes with the effect estimates, since studies that used different time windows were included. This analysis showed again no evidence of subgroup differences, although the effect estimates were more prominent for the efficacy outcomes when the cut-off was set at 7 days, possibly due to larger sample size.

Determining the optimal timing of OAC initiation in patients with acute ischemic stroke and atrial fibrillation is contingent upon individual patient factors. Strict adherence to predefined early or late initiation thresholds in general guidelines may oversimplify the complexities inherent in stroke prevention for this patient population. For instance, patients with an increased bleeding risk, such as those with a history of hemorrhagic transformation, severe or large stroke, or advanced age might derive greater benefit from delayed OAC initiation.^25,26^ However, analyses of prespecified subgroups of the ELAN and TIMING population, assessing for different age, stroke severity and infarct size, did not disclose any statistically significant differences regarding the treatment effect of early versus late OAC initiation.^8,9^ Similarly, when real-world data are considered, the associations between OAC initiation timing and clinical outcomes were not affected by either infarct size or hemorrhagic transformation, as shown in the sensitivity analyses of Yaghi et al.^24^ Yet, these were exploratory analyses aimed at generating hypotheses rather than establishing treatment effects. On the other hand, contradictory observational data also exist, suggesting a higher risk of intracranial hemorrhage among patients with large infarctions.^18^ Moreover, optimizing treatment decisions necessitates a comprehensive clinical assessment encompassing comorbidities (including prior atrial fibrillation history), polypharmacy, and frailty, alongside a collaborative approach involving specialists such as geriatricians.^27^ Particularly concerning frail patients, extrapolating findings from studies conducted in other populations might not yield the anticipated results. For example, the Switching anticoagulant management from a vitamin K antagonist to a NOAC-based treatment strategy in frail elderly patients with atrial fibrillation (FRAIL-AF) study found that switching from vitamin K antagonists to NOACs was associated with a higher bleeding risk, yet similar thromboembolic risk, compared to continuing vitamin K antagonists, likely due to close monitoring of the international normalized ratio and individualized adjustments in the vitamin K antagonist-group.^28^ Furthermore, along with OAC initiation, addressing modifiable risk factors for both bleeding and stroke recurrence, such as arterial hypertension, is crucial and should not be underestimated.^29,30^ Consequently, the evaluation of the potential interactions between timing and patient characteristics requires a more nuanced approach. A future individual patient data meta-analysis holds promise in this regard. By synthesizing data at the individual patient level from various RCTs, such an analysis can provide more precise insights into the impact of timing on outcomes across different patient profiles.

Our current systematic review and meta-analysis serve to complement the results of a previous study conducted by our group.^7^ In our prior work, we conducted a pooled analysis of mostly observational data, demonstrating comparable efficacy and safety between patients undergoing early versus late OAC initiation. Expanding upon this groundwork, the incorporation of recently published high-quality RCT data in our current systematic review allowed for a larger sample size and facilitated a direct comparative meta-analysis. Consequently, our present findings not only reaffirm similar safety outcomes but also indicate enhanced efficacy in terms of reducing the composite outcome and ischemic stroke recurrence among patients with early compared to late OAC initiation. Furthermore, according to our previous meta-analysis, initiating NOACs as opposed to vitamin K antagonists was associated with lower rates of ischemic stroke recurrence, either in the early or in the late window of initiation.^7^ Indeed, existing guidelines support the use of NOACs over vitamin K antagonists, when anticoagulant treatment is considered for the management of non-valvular atrial fibrillation due to similar efficacy and more favorable safety profile of NOACs.^4^ Based on these notions, we performed a sensitivity analysis by including only those studies that specifically evaluated patients initiating NOACs. This analysis confirmed the same results apart from ischemic stroke recurrence, for which the point estimate was in favor of early NOAC initiation, yet without being statistically significant. This fragile result, as unraveled by the sensitivity analysis, underscores the need for further high-quality data that are expected from ongoing RCTs.

To that aim, two RCTs are currently ongoing: the Optimal delay time to initiate anticoagulation after ischemic stroke in atrial fibrillation (START) trial (NCT03021928) and the OPtimal TIMing of Anticoagulation After Acute Ischemic Stroke (OPTIMAS) trial (NCT03759938). In the START trial, conducted in United States, 1500 acute ischemic stroke patients with atrial fibrillation were randomized to initiate NOACs in four distinct time windows stratified by stroke severity (i.e. mild or moderate and high-risk stroke).^31^ More specifically, patients with mild or moderate-risk strokes were randomized in initiating NOACs within 3, 6, 10, or 14 days post acute ischemic stroke, while patients with high-risk stroke were randomized in initiating NOACs within 6, 10, 14, or 21 days post acute ischemic stroke. It is important to note that patients that were complicated with symptomatic intracranial hemorrhage (those patients were excluded from previous RCTs) were able to participate as high-risk stroke patients in the START trial, providing valuable information for this vulnerable patient subgroup. While recruitment for the START trial has concluded, the study results have not yet been announced and are eagerly anticipated. The OPTIMAS trial, conducted in United Kingdom, is expected to randomize more than 3000 acute ischemic stroke patients with atrial fibrillation in early NOAC initiation (early defined as within 4 days post acute ischemic stroke) versus later initiation (7–14 days post acute ischemic stroke).^32^ This trial is designed using a non-inferiority gatekeeper analysis approach with a nested test of superiority, similarly to the TIMING trial.^9^ Another important aspect of the OPTIMAS trial is the fact that patients that were already anticoagulated at stroke onset or were complicated with a parenchymal hematoma type 1 were also allowed for inclusion, in contrast to the ELAN trial that excluded these specific patient subgroups.^8^ Furthermore, in contrast to the ELAN trial that accounted for index infarct size, OPTIMAS follows a more “one-size-fits-all” approach, expected to answer whether OAC initiation as early as within 4 days may be feasible and safe in patients with acute ischemic stroke of larger size. Importantly, according to the trial sequential analysis that we conducted, an additional 812 patients from a future RCT are needed to provide a more robust result for the composite outcome. This required information size is expected to be easily fulfilled by the ongoing RCTs, hopefully providing more definitive answers to the clinical conundrum of OAC initiation.

Since the publication of the results of the ELAN trial,^8^ several systematic reviews and meta-analyses were performed with the aim to evaluate the efficacy and safety of early versus later OAC initiation. One of those included 12 studies (2 RCTs and 10 observational studies),^33^ but it should be noted that some of the included observational s tudies were duplicated cohorts, for example, the cohort from Erlangen/Germany^34^ which was already included in the study of De Marchis et al.^19^ Similarly, in another systematic review and meta-analysis,^35^ the CROMIS-2 study was included,^36^ which was already part of the individual patient-data pooled analysis of De Marchis et al.^19^ Although the main results of the aforementioned studies align with the findings of our meta-analysis, the inclusion of duplicate data could have potentially compromised their quality. In our study, not only did we ensure that no duplicate data were included, but two prespecified subgroup analyses were performed to assess potential sources of heterogeneity. Sensitivity and trial sequential analysis further enhanced the completeness of our methodology.

Despite those strengths of our study, there are certain limitations that need to be acknowledged. First, our meta-analysis included data from observational studies which occupied the majority of the study-weights during syntheses. Although most baseline characteristics, including age, sex, CHA_2_DS_2_VASc, and HASBLED scores, proportion of receiving acute reperfusion therapies, and proportion of small infarct acute ischemic stroke were similar between the compared groups, the mean NIHSS score was significantly lower in the patients that initiated OACs early versus later, highlighting a potential selection bias for treatment initiation among the observational studies. Unfortunately, considering that the included studies were limited (less than 10), a meta-regression analysis could not be pursued in order to further explore whether NIHSS mean difference was a significant moderator of the study outcomes. Importantly, when analyses were restricted to RCTs only, that presented no baseline imbalances, similar results to the main analysis were obtained. Second, the included studies did not present uniformity regarding the time-window that was used for the definition of early initiation. We addressed this shortcoming by conducting subgroup analyses based on the different treatment time windows, showing that initiating OACs either within the first 7 days or even earlier was similarly effective and safe. Furthermore, although we have conducted a sensitivity analysis focusing on patients that exclusively initiated NOACs (rather than vitamin K antagonists), further data on potential differences between specific NOAC regimens or their different doses (full vs reduced doses) were not available. Finally, publication bias were uncovered during evaluation for the efficacy outcomes, possibly due to small study effects. This limitation is expected to be diminished, when larger RCTs conclude and present their results, allowing for both high-quality, study-level meta-analysis, and individual patient-data meta-analysis, that has already been planned under a common collaboration.

## Conclusions

In conclusion, this updated meta-analysis confirms similar safety of early OAC initiation in acute ischemic stroke patients with atrial fibrillation compared to later OAC initiation, while, for the first time, marginal superiority of early OAC initiation is shown, in terms of the reduction of the composite outcome and of ischemic stroke recurrence at follow-up.

## Supplemental Material

sj-docx-1-eso-10.1177_23969873241251931 – Supplemental material for Timing of oral anticoagulants initiation for atrial fibrillation after acute ischemic stroke: A systematic review and meta-analysis Supplemental material, sj-docx-1-eso-10.1177_23969873241251931 for Timing of oral anticoagulants initiation for atrial fibrillation after acute ischemic stroke: A systematic review and meta-analysis by Lina Palaiodimou, Maria-Ioanna Stefanou, Aristeidis H Katsanos, Gian Marco De Marchis, Diana Aguiar De Sousa, Jesse Dawson, Mira Katan, Theodore Karapanayiotides, Konstantinos Toutouzas, Maurizio Paciaroni, David J Seiffge and Georgios Tsivgoulis in European Stroke Journal

sj-docx-2-eso-10.1177_23969873241251931 – Supplemental material for Timing of oral anticoagulants initiation for atrial fibrillation after acute ischemic stroke: A systematic review and meta-analysis Supplemental material, sj-docx-2-eso-10.1177_23969873241251931 for Timing of oral anticoagulants initiation for atrial fibrillation after acute ischemic stroke: A systematic review and meta-analysis by Lina Palaiodimou, Maria-Ioanna Stefanou, Aristeidis H Katsanos, Gian Marco De Marchis, Diana Aguiar De Sousa, Jesse Dawson, Mira Katan, Theodore Karapanayiotides, Konstantinos Toutouzas, Maurizio Paciaroni, David J Seiffge and Georgios Tsivgoulis in European Stroke Journal
