# Supplementary material for: Timing of oral anticoagulants initiation for atrial fibrillation after acute ischemic stroke: A systematic review and meta-analysis
Source: Eur Stroke J. 2024 May 14;9(4):885–95. doi: 10.1177/23969873241251931 (PMC11569516; doi:10.1177/23969873241251931)

**eFigures:**

**eFigures 1-26**

**eFigure 1.** Summary plot presenting the quality assessment of included randomized controlled clinical trials using the Cochrane Collaboration tool (RoB 2).


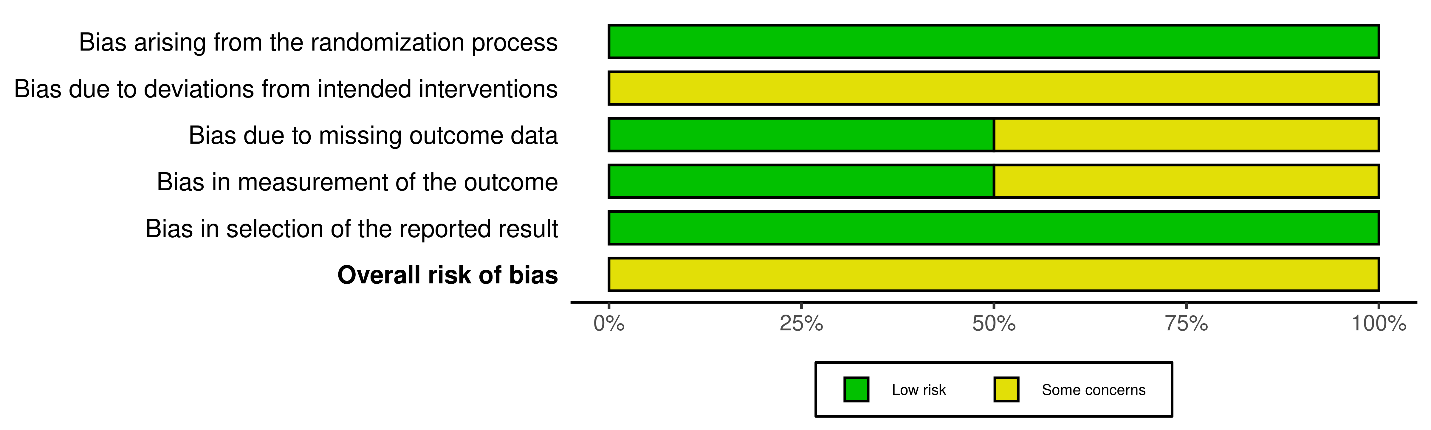


**eFigure 2.** Traffic Light Plot presenting the quality assessment of included randomized controlled clinical trials using the Cochrane Collaboration tool (RoB 2).


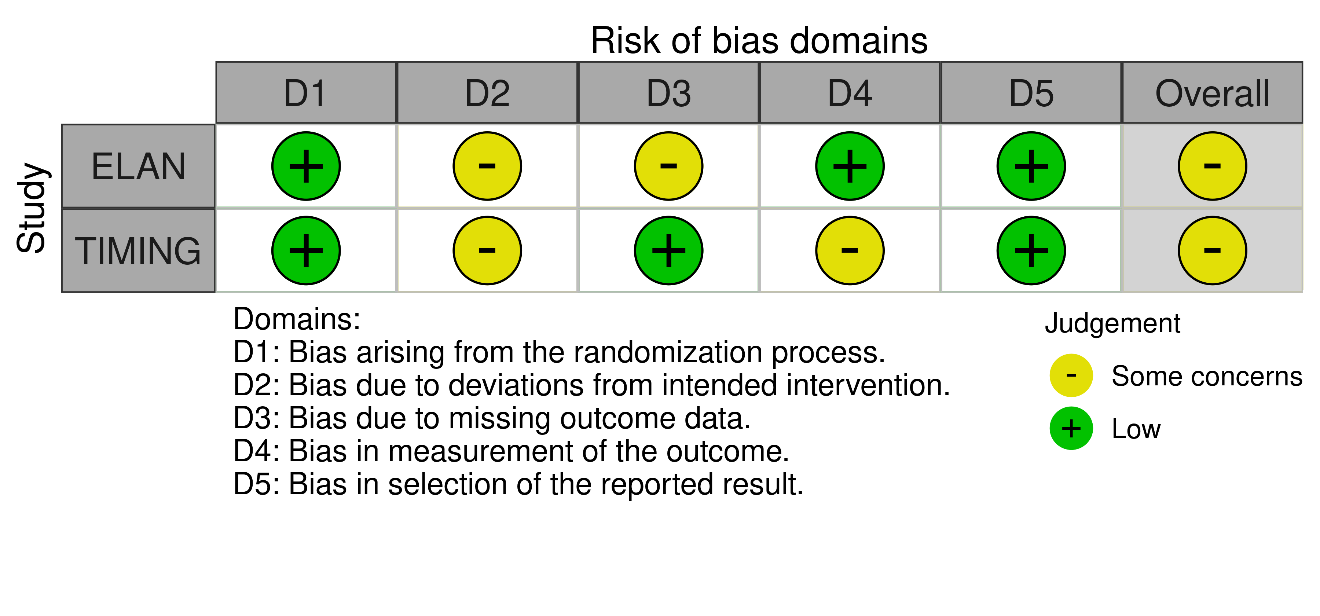


**eFigure 3.** Summary plot presenting the quality assessment of included observational studies using the Risk Of Bias In Non-randomized Studies of Interventions (ROBINS-I) tool.


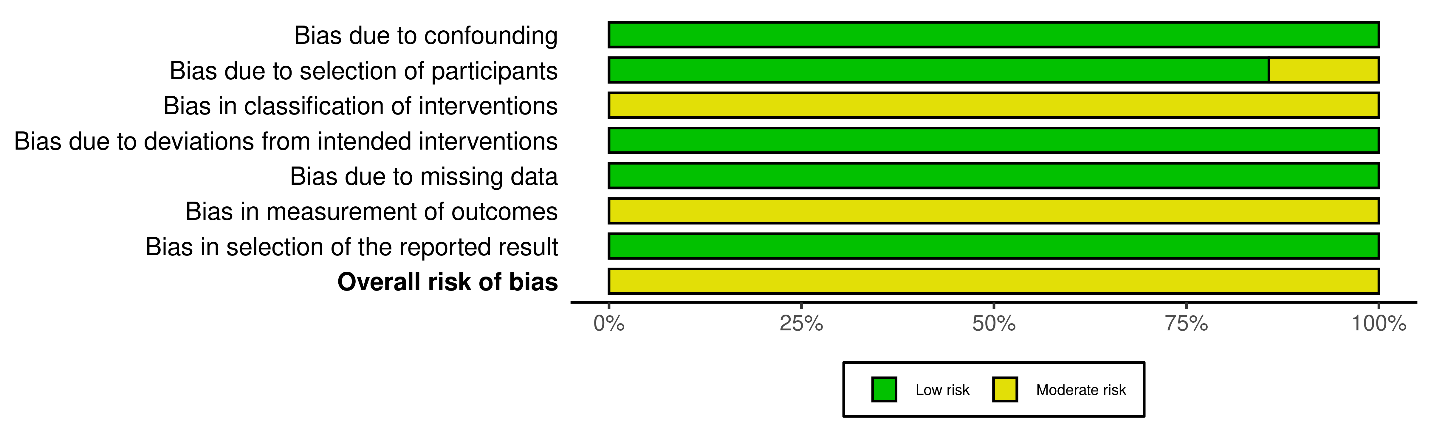


**eFigure 4.** Traffic Light Plot presenting the quality assessment of included observational studies using the Risk Of Bias In Non-randomized Studies of Interventions (ROBINS-I) tool.


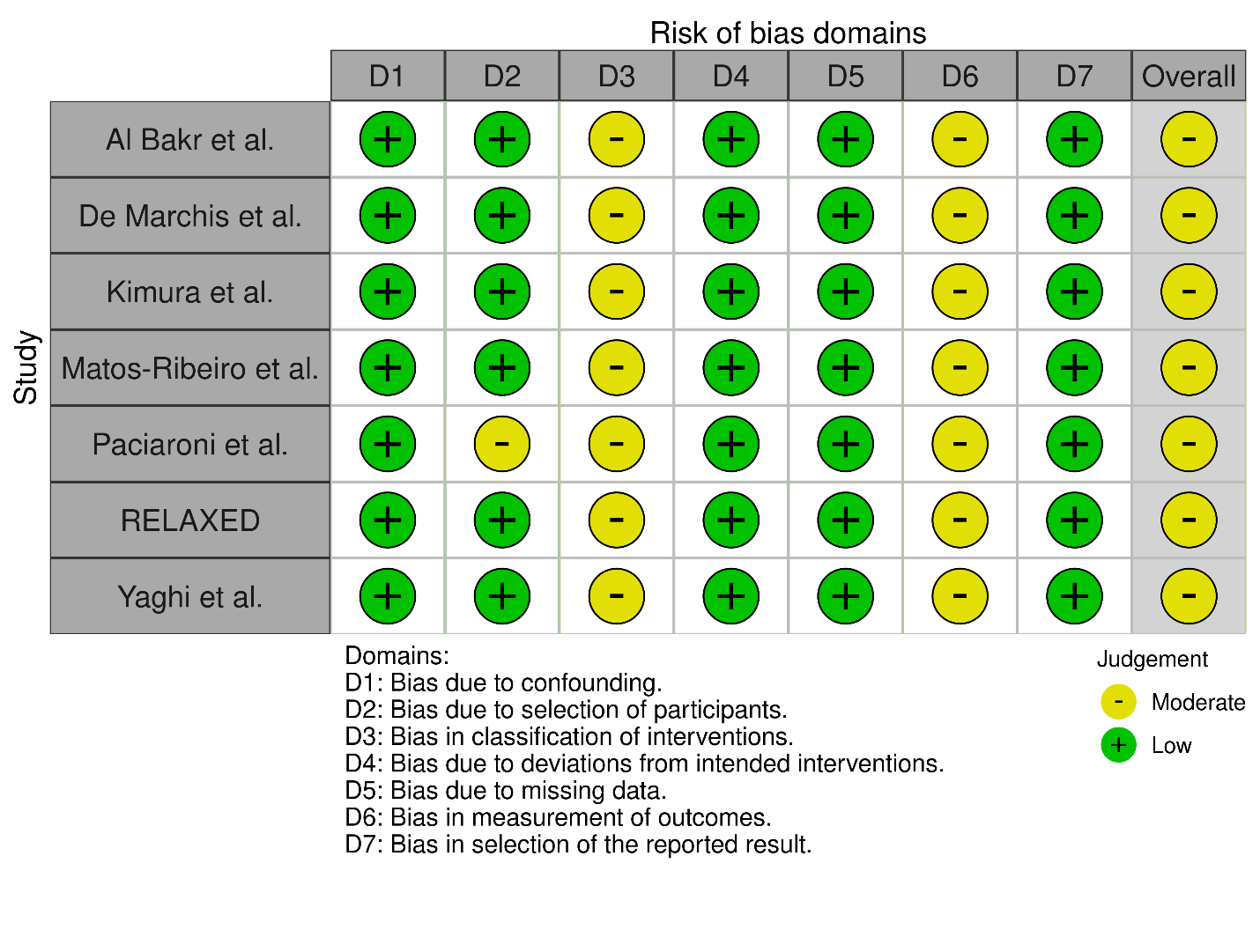


**eFigure 5.** Forest plot presenting the mean difference of age (in years) among the patients in the early versus late group of OAC initiation.


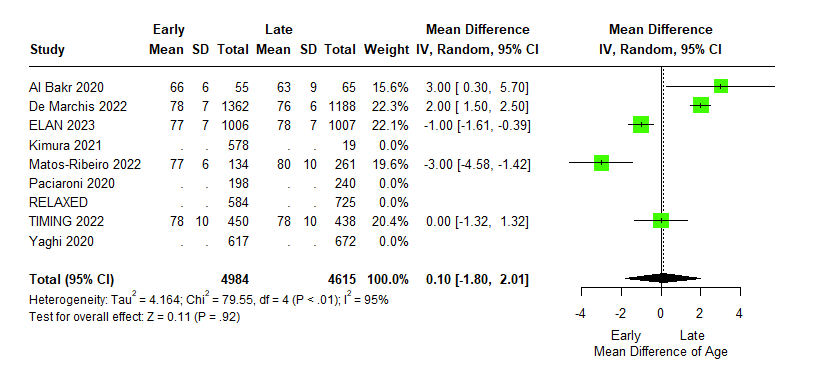


**eFigure 6.** Forest plot presenting the odds ratio of women among the patients in the early versus late group of OAC initiation.


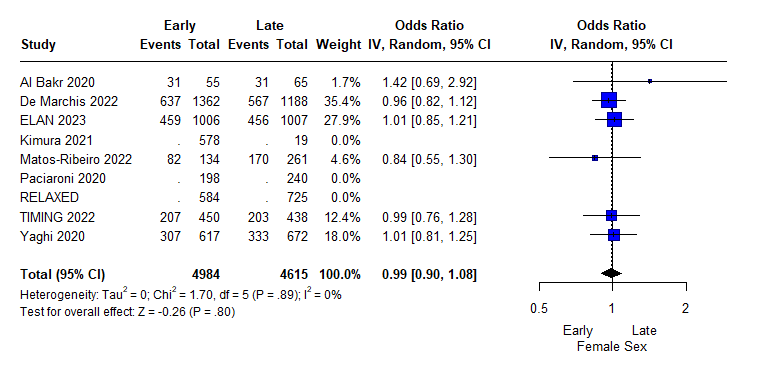


**eFigure 7.** Forest plot presenting the mean difference of CHA_2_DS_2_VASc score among the patients in the early versus late group of OAC initiation.


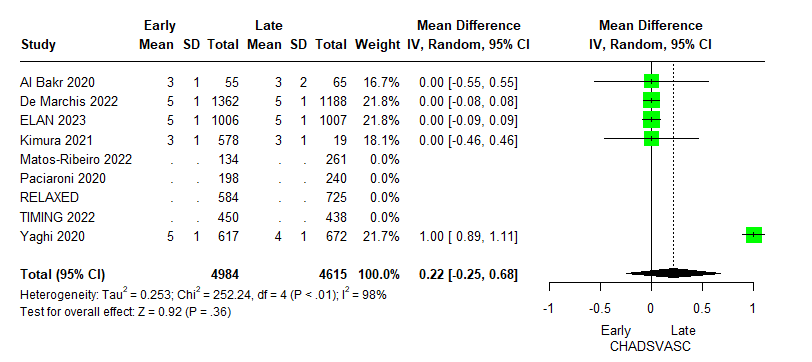


**eFigure 8.** Forest plot presenting the mean difference of HASBLED score among the patients in the early versus late group of OAC initiation.


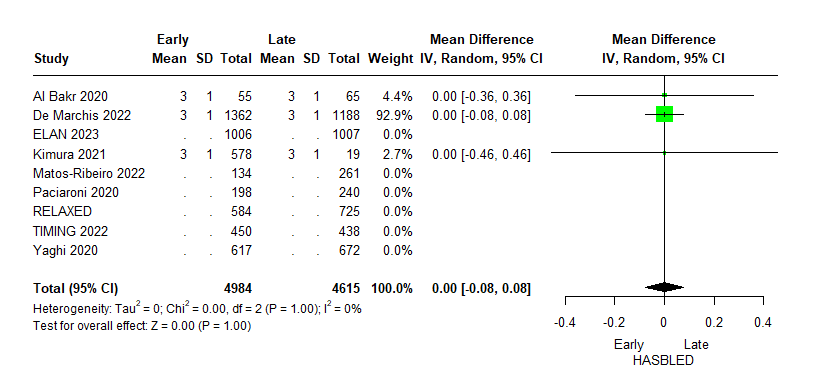


**eFigure 9.** Forest plot presenting the mean difference of NIHSS score among the patients in the early versus late group of OAC initiation.


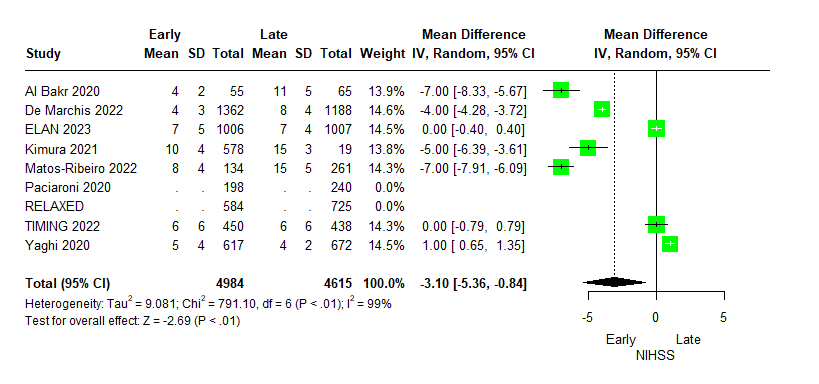


**eFigure 10.** Forest plot presenting the odds ratio of patients receiving acute reperfusion treatment among the patients in the early versus late group of OAC initiation.


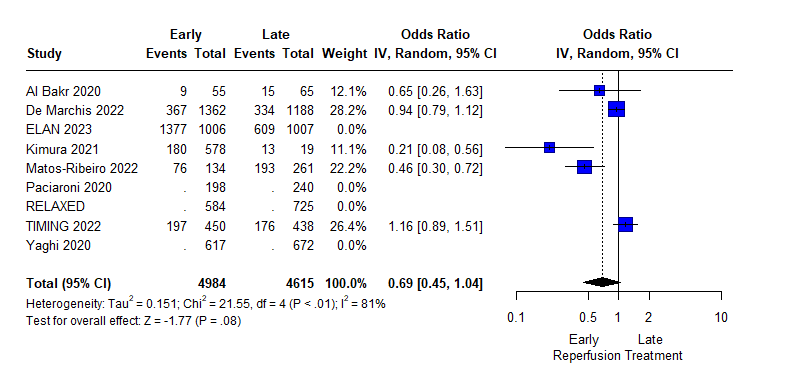


**eFigure 11.** Forest plot presenting the odds ratio of patients with small infarct acute ischemic stroke among the patients in the early versus late group of OAC initiation.


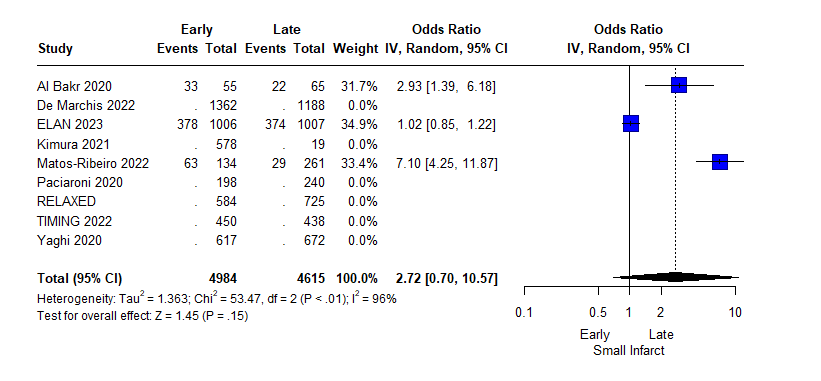


**eFigure 12.** Subgroup analysis stratified by different time windows for early initiation: forest plot presenting the risk ratio of the composite outcome at follow-up among the patients in the early versus late group of NOAC initiation (p for subgroup differences=0.0523).


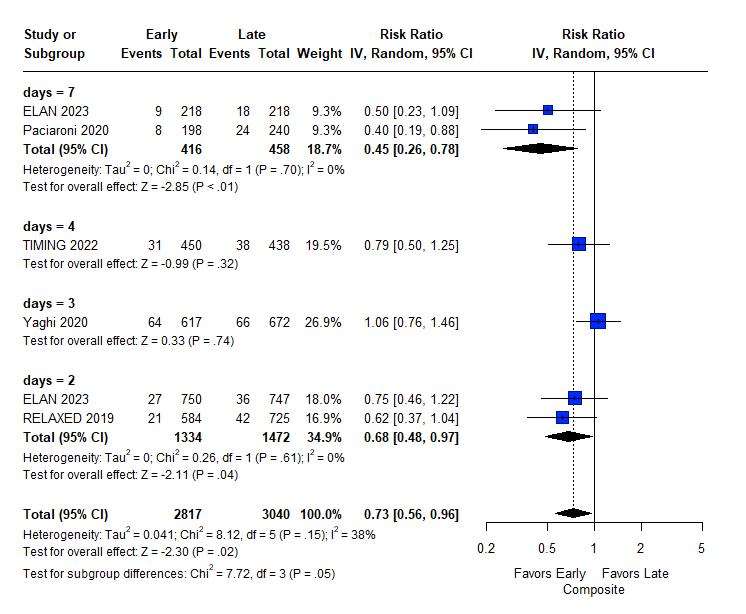


**eFigure 13.** Subgroup analysis stratified by different time windows for early initiation: forest plot presenting the risk ratio of ischemic stroke recurrence at follow-up among the patients in the early versus late group of NOAC initiation.


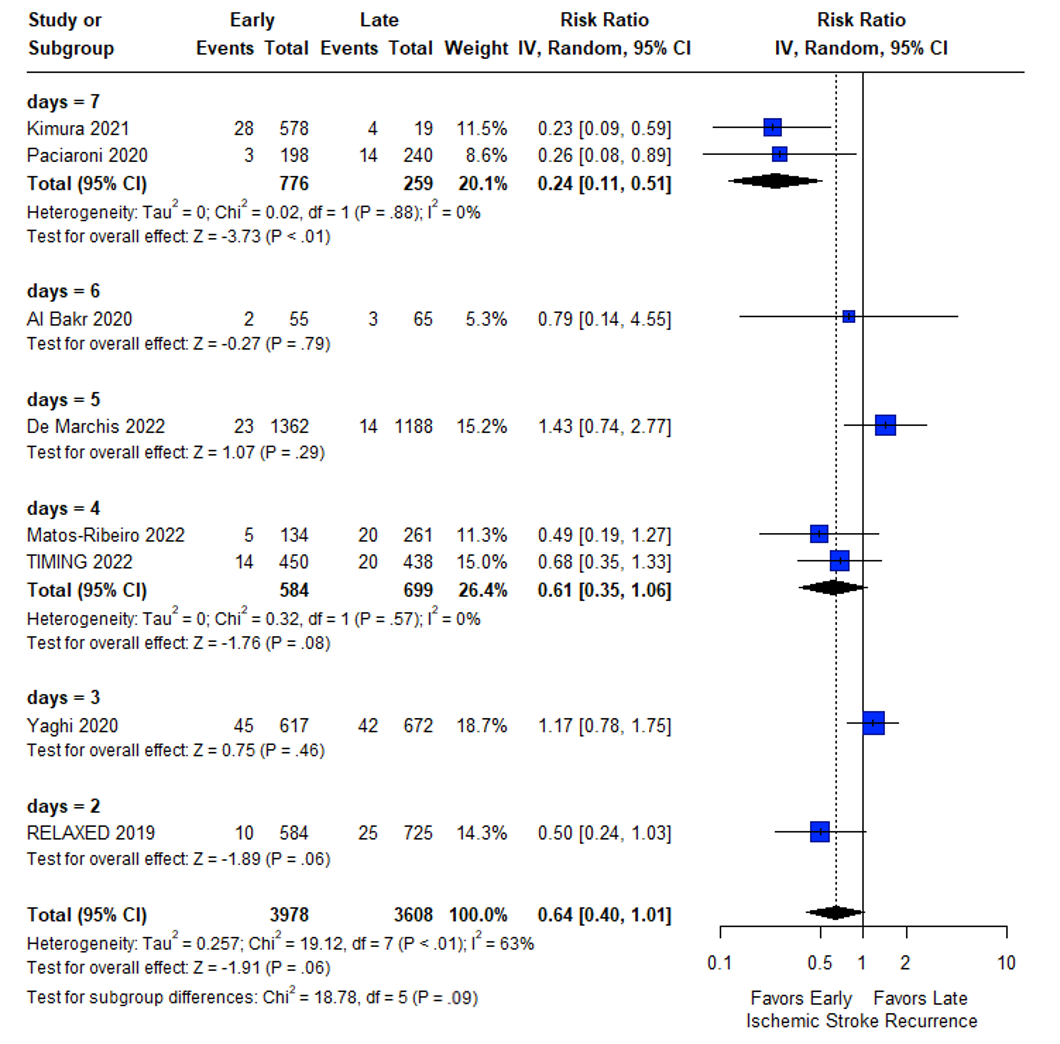


**eFigure 14.** Subgroup analysis stratified by different time windows for early initiation: forest plot presenting the risk ratio of intracranial hemorrhage at follow-up among the patients in the early versus late group of NOAC initiation.


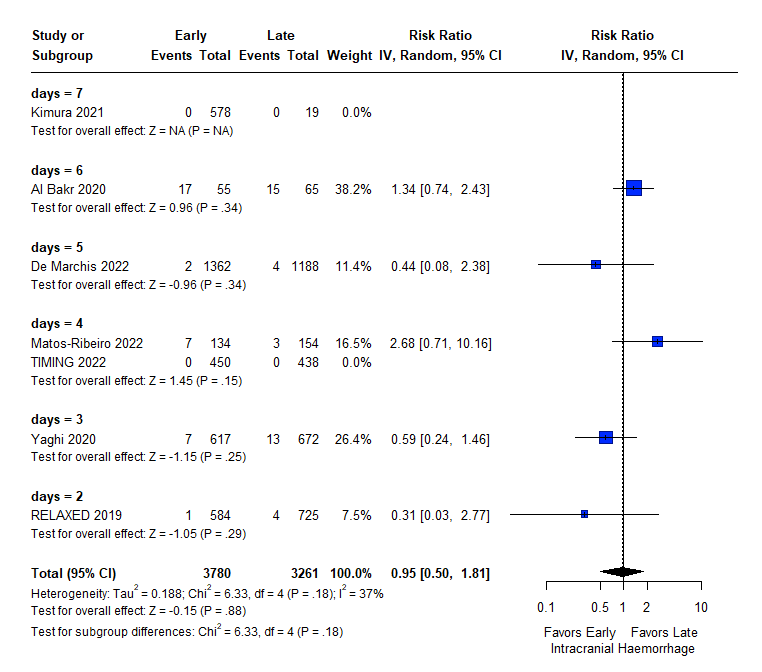


**eFigure 15.** Subgroup analysis stratified by different time windows for early initiation: forest plot presenting the risk ratio of major bleeding at follow-up among the patients in the early versus late group of NOAC initiation.


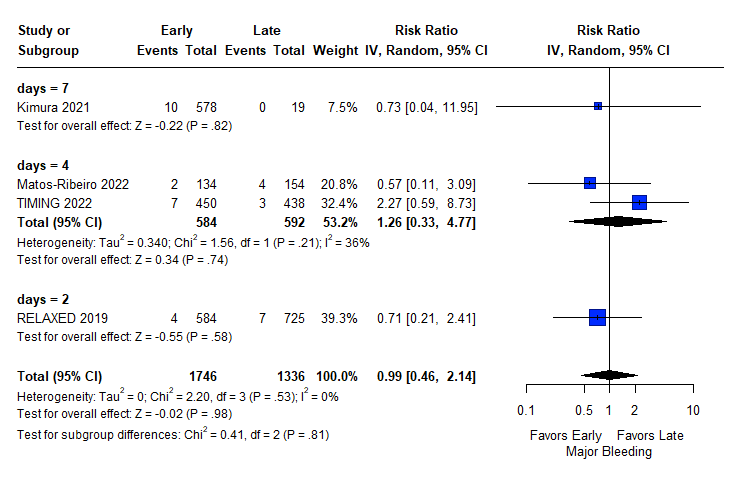


**eFigure 16.** Subgroup analysis stratified by different time windows for early initiation: forest plot presenting the risk ratio of all-cause mortality at follow-up among the patients in the early versus late group of NOAC initiation.


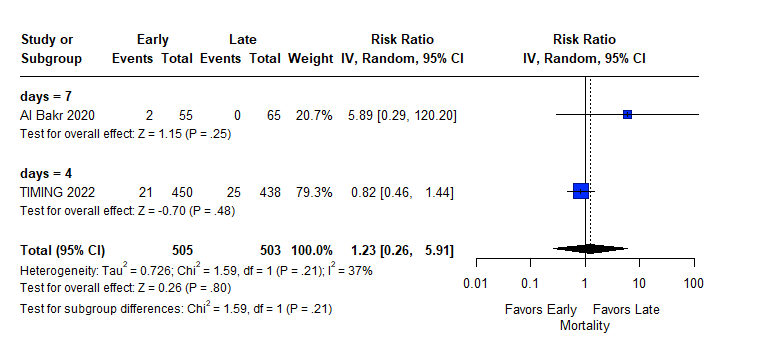


**eFigure 17.** Sensitivity analysis restricted to the studies that included patients specifically initiating NOACs. Forest plot presenting the risk ratio of the composite outcome at follow-up among the patients in the early versus late group of NOAC initiation.


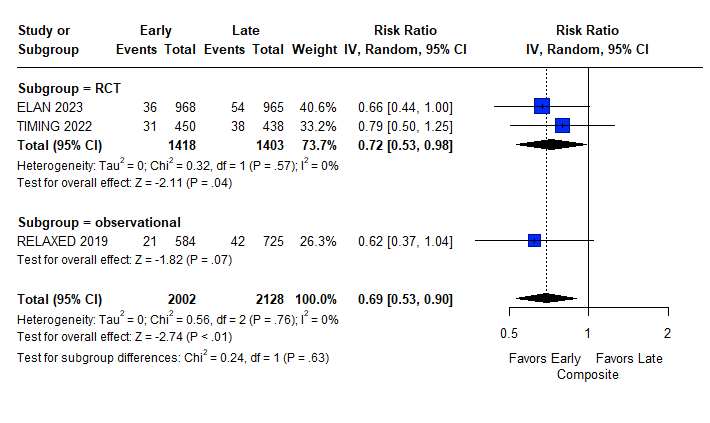


**eFigure 18.** Sensitivity analysis restricted to the studies that included patients specifically initiating NOACs. Forest plot presenting the risk ratio of ischemic stroke recurrence at follow-up among the patients in the early versus late group of NOAC initiation.


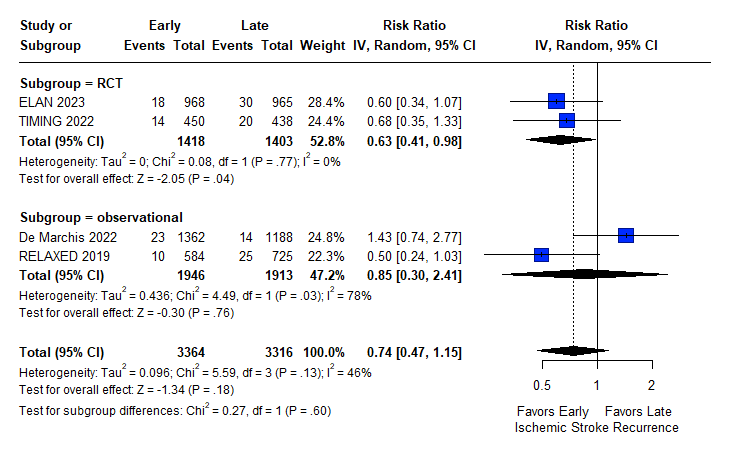


**eFigure 19.** Sensitivity analysis restricted to the studies that included patients specifically initiating NOACs. Forest plot presenting the risk ratio of intracranial hemorrhage at follow-up among the patients in the early versus late group of NOAC initiation.


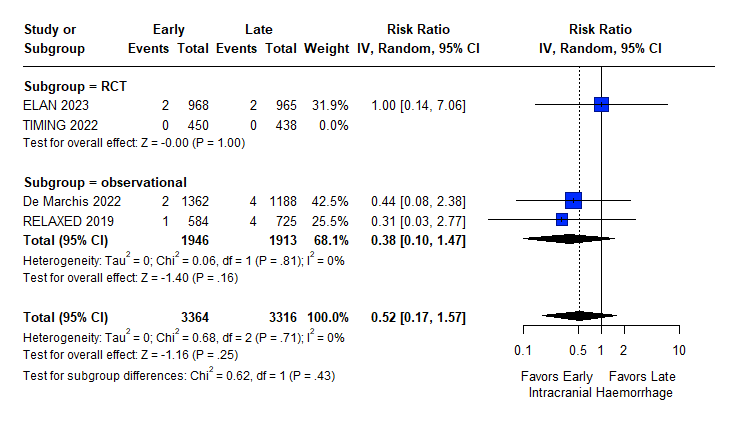


**eFigure 20.** Sensitivity analysis restricted to the studies that included patients specifically initiating NOACs. Forest plot presenting the risk ratio of major bleeding at follow-up among the patients in the early versus late group of NOAC initiation.


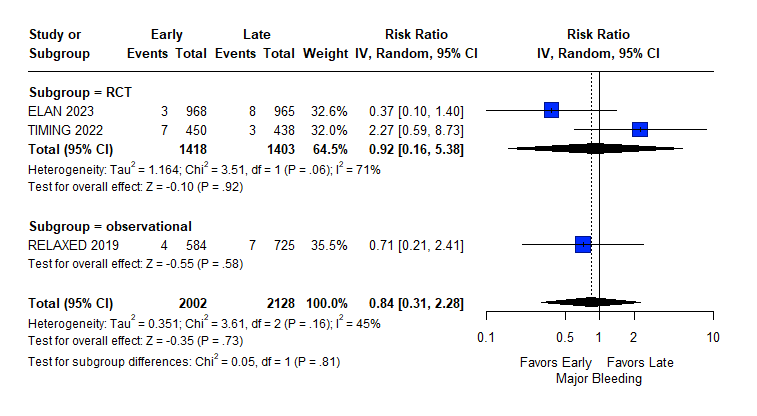


**eFigure 21.** Sensitivity analysis restricted to the studies that included patients specifically initiating NOACs. Forest plot presenting the risk ratio of all-cause mortality at follow-up among the patients in the early versus late group of NOAC initiation.


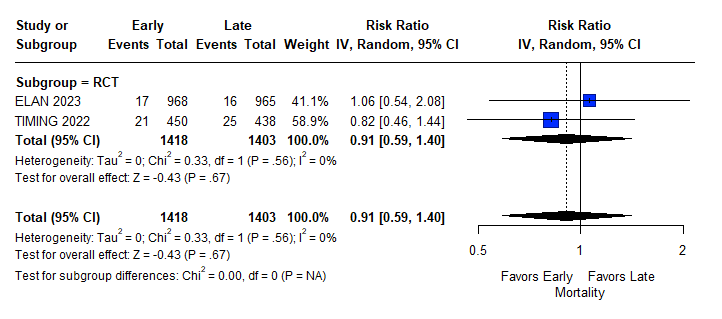


**eFigure 22.** Funnel plot on the reported rates of the composite outcome (p for Egger's test= 0.0331).


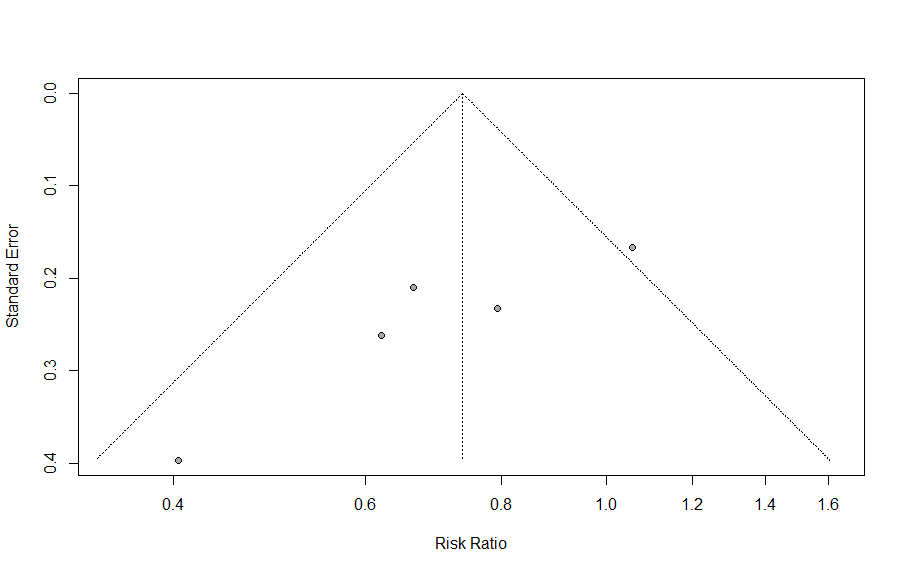


**eFigure 23.** Funnel plot on the reported rates of ischemic stroke recurrence (p for Egger's test= 0.0680).


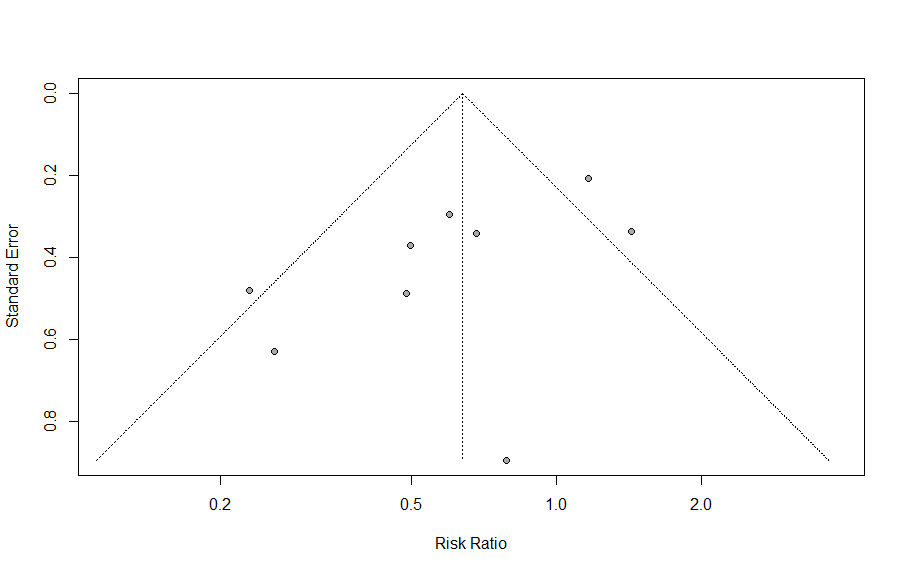


**eFigure 24.** Funnel plot on the reported rates of intracranial hemorrhage (p for Egger's test= 0.4261).


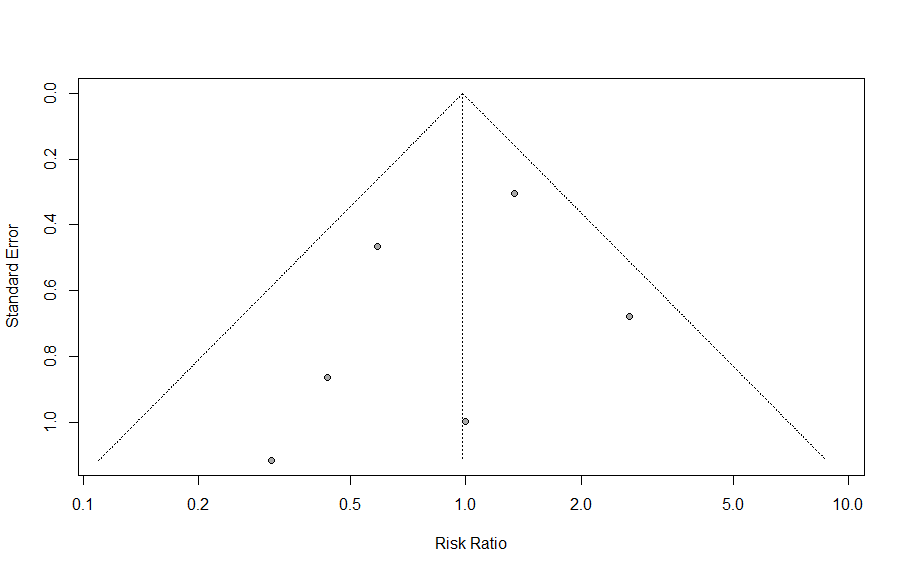


**eFigure 25.** Funnel plot on the reported rates of major bleeding (p for Egger's test= 0.9257).


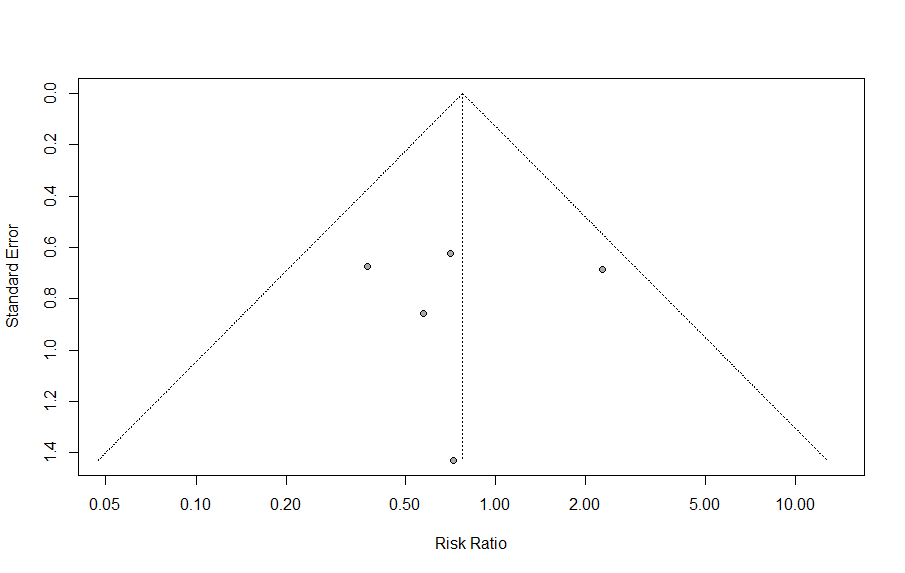


**eFigure 26.** Trial sequential analysis for the composite outcome among included randomized-controlled clinical trials. The last point of Z-curve is outside the conventional test boundary but within the monitoring boundaries, while the inner wedge is not reached. The required information size has been calculated at 3,633 patients, i.e., an additional 812 patients from a future RCT.


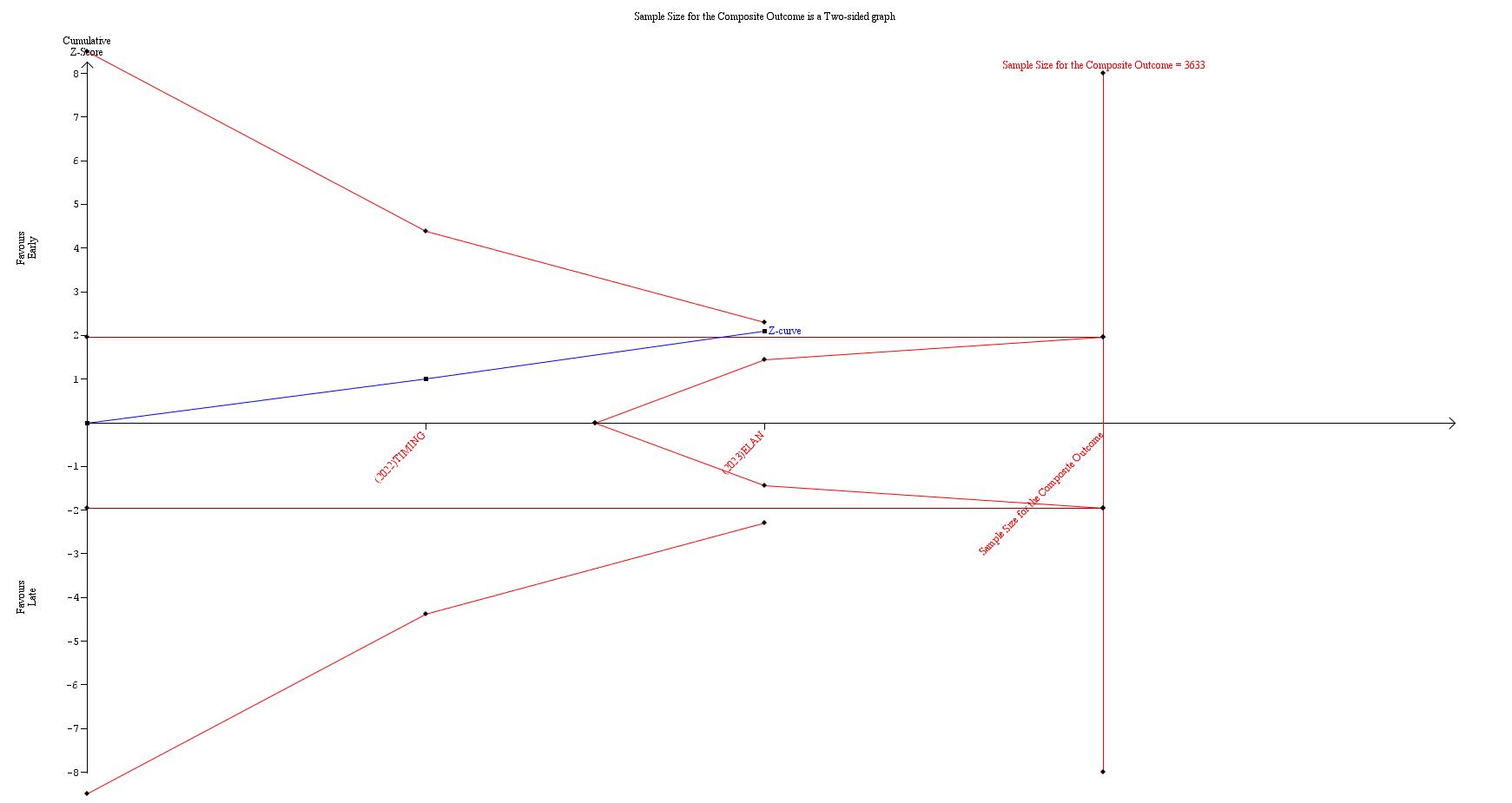

Supplement: sj-docx-2-eso-10.1177_23969873241251931 – Supplemental material for Timing of oral anticoagulants initiation for atrial fibrillation after acute ischemic stroke: A systematic review and meta-analysis [file sj-docx-2-eso-10.1177_23969873241251931.docx]
